# Knowledge-based radiation therapy (KBRT) treatment planning versus planning by experts: validation of a KBRT algorithm for prostate cancer treatment planning

**DOI:** 10.1186/s13014-015-0416-6

**Published:** 2015-05-10

**Authors:** Obioma Nwankwo, Hana Mekdash, Dwi Seno Kuncoro Sihono, Frederik Wenz, Gerhard Glatting

**Affiliations:** Department of Radiation Oncology, Universitätsmedizin Mannheim, Medical Faculty Mannheim, Heidelberg University, Mannheim, Germany; Medical Radiation Physics/Radiation Protection, Universitätsmedizin Mannheim, Medical Faculty Mannheim, Heidelberg University, Mannheim, Germany

**Keywords:** Knowledge-based radiation therapy (KBRT) treatment planning, Personalized radiotherapy treatment planning, Dose prediction algorithm, Treatment plan optimization, Normal tissue sparing

## Abstract

**Background:**

A knowledge-based radiation therapy (KBRT) treatment planning algorithm was recently developed. The purpose of this work is to investigate how plans that are generated with the objective KBRT approach compare to those that rely on the judgment of the experienced planner.

**Methods:**

Thirty volumetric modulated arc therapy plans were randomly selected from a database of prostate plans that were generated by experienced planners (*expert plans*)*.* The anatomical data (CT scan and delineation of organs) of these patients and the KBRT algorithm were given to a novice with no prior treatment planning experience. The inexperienced planner used the knowledge-based algorithm to predict the dose that the OARs receive based on their proximity to the treated volume. The population-based OAR constraints were changed to the predicted doses. A *KBRT plan* was subsequently generated. The *KBRT* and *expert* plans were compared for the achieved target coverage and OAR sparing. The target coverages were compared using the Uniformity Index (UI), while 5 dose-volume points (D_10_, D_30,_ D_50_, D_70_ and D_90_) were used to compare the OARs (bladder and rectum) doses. Wilcoxon matched-pairs signed rank test was used to check for significant differences (p < 0.05) between both datasets.

**Results:**

The KBRT and expert plans achieved mean UI values of 1.10 ± 0.03 and 1.10 ± 0.04, respectively. The Wilcoxon test showed no statistically significant difference between both results. The D_90_, D_70,_ D_50_, D_30_ and D_10_ values of the two planning strategies, and the Wilcoxon test results suggests that the KBRT plans achieved a statistically significant lower bladder dose (at D_30_), while the expert plans achieved a statistically significant lower rectal dose (at D_10_ and D_30_).

**Conclusions:**

The results of this study show that the KBRT treatment planning approach is a promising method to objectively incorporate patient anatomical variations in radiotherapy treatment planning.

## Background

Inverse treatment planning is now a standard treatment planning method and it normally starts with a template, which aids the specification of the planning objectives. Templates contain population-based experience of the constraints that are suitable for planning the dose delivery to a particular treatment region. The treatment planner sometimes modifies these constraints to account for anatomical variations amongst patients. This modification is based on the planner’s subjective judgment of the constraints that are suitable for planning the given anatomy.

Knowledge-based radiation therapy treatment (KBRT) is a technique to objectively incorporate prior experience into radiotherapy treatment planning. KBRT has been proposed as a method of transferring knowledge from the experienced to the less experienced institutions [1]. Recently, several KBRT approaches [2–8] have been described. Our algorithm [2] is derived from the analysis of treatment plans optimized by experts. The algorithm relates the dose that is received by the organs-at-risk (OARs) to their geometric proximity to the treated volume, and thus provides a method to objectively incorporate individual patient anatomical variations in the treatment planning and acceptance process. Unlike the other approaches, the algorithm can predict the 3D dose distribution in the organs of interest.

The likelihood of radiation therapy treatment planning becoming fully automated in the next ten years was recently debated [9]. The opponent of this proposition conceded that KBRT and/or computer-aided multicriterial optimization would be widely employed to eliminate the present-day human variability from the treatment plan optimization process [9]. But before KBRT can be confidently adopted as a clinical tool, whether to aid or to replace the experienced planner as argued in the cited work, it is necessary to compare the quality of plans that are generated with the KBRT approach against those that are made by experienced planners.

Therefore, the purpose of this study is to evaluate how this objective approach compares to the subjective judgment of the experienced treatment planner in terms of treatment plan quality. A novice planner with no previous treatment planning experience was given the anatomical data of 30 subjects that were treated for prostate cancer in our institution. The inexperienced planner utilized the KBRT algorithm to predict the probable dose of the OARs based on their proximity to the target volume. The predicted information was thereafter used to modify the reference constraints and a plan was subsequently generated. These plans were compared to the reference plans that were made by experienced planners.

## Methods

### Ethics approval and patient selection

This study was approved by the Medical Ethics Commission of the Medical Faculty Mannheim, Heidelberg University. Thirty volumetric modulated arc therapy (VMAT) plans were randomly selected from a database of patients that were treated for prostate cancer at the University Medical Centre, Mannheim. These *expert plans* were created by experienced planners. An inexperienced planner, aided by a KBRT algorithm [2] generated a second set of *KBRT plans* for the 30 subjects. All the plans were made with the *MONACO®* treatment planning system (CMS, Elekta, Crawley, UK). The following sections detail how these plans were generated.

#### Expert plans

The expert plans were created by experienced planners and were approved by a physician and a physicist for patient treatment. We define an experienced planner as personnel who is knowledgeable in treatment planning by virtue of a good understanding of the treatment planning system and the planning constraints that are suitable/applicable for planning a given treatment site. The making of these plans began with a template to aid the specification of the planning constraints. Experienced planners created the templates that are used in our institution. A template, which is used for prostate cancer planning in our institution, is shown in Table 1. The planner sometimes modifies these reference constraints (for the dose to the structures of interest). For example, a planner may reduce or increase the reference (template-specified) dose to the bladder based on the fraction of this organ that is included in the planning target volume (PTV). If the initial plan that is generated (with or without the modification of the reference constraints) is deemed to be of poor quality, the planning constraints are further adjusted and the case is replanned. This process is repeated until a plan is generated which is acceptable to both the responsible physician and physicist. This iterative process is both time-consuming and labor intensive.

**Table 1:** A MONACO® planning template for prostate VMAT at the Universitätsmedizin Mannheim. P + SB = prostate and seminal vesicles

Structure	Cost function	Reference dose (cGy)	Isoconstraint
P + SB	Target EUD		6000.0
	Quadratic overdose	6000.0	70.0
	Quadratic underdose	6000.0	70.0
Bladder	Parallel	3000.0	40.0
	Serial		3500.0
Rectum	Parallel	3000.0	30.0
	Quadratic overdose	3000.0	50.0
Patient	Quadratic overdose	3500.0	120.0
	Quadratic overdose	2200.0	60.0
	Conformality		0.7

#### KBRT plans

The KBRT algorithm and planning CT (the input to the algorithm) were given to a novice with no prior treatment planning experience. The inexperienced planner used the algorithm to predict the likely dose to the OARs (rectum and bladder) based on their proximity to the PTV [2]. Dose-volume constraints extracted from the predicted 3D dose distribution were used to modify the reference constraints of the template and a KBRT plan was subsequently generated. Hence, all other planning parameters (i.e. beam arrangement and dose calculation properties) were the same for the KBRT and expert plans.

#### Comparison of the quality of the KBRT and expert plans

Both sets of plans were compared for the achieved target coverage and normal tissue sparing. The uniformity index (UI) provided a quantitative measure of the target coverage. The UI is defined as the ratio of D_05_ and D_95_

1 $$ UI=\frac{D_{05}}{D_{95}} $$

where D_05_ and D_95_ are the maximum doses that cover at least 5 % and 95 % of the target volumes respectively. UI values closer to 1 indicate better homogeneity, while larger values imply increasing heterogeneity [10, 11]. To compare the planned doses to the OARs, five dose-volume points (D_x_ = D_90_, D_70,_ D_50_, D_30_ and D_10_) were used, where D is the maximum dose that is received by x % volume of the organ. The dose difference DD_*x*_, between the individual D_*x*_ values of the KBRT and expert plans were also calculated according to

2 $$ D{D}_x={D}_x^{KBRT}-{D}_x^{Expert} $$

#### Statistical analysis

Mean values and standard deviations were calculated for descriptive statistics. Wilcoxon matched-pairs signed rank tests were performed using GraphPad Prism (version 6.05 for Windows, GraphPad Software, La Jolla California USA, www.graphpad.com). P values lower than 0.05 were considered to denote statistically significant differences between the compared datasets.

## Results

### Target coverage

The KBRT plans achieved an average Uniformity Index (UI) of 1.10 ± 0.04, while the expert plans achieved an average UI of 1.10 ± 0.03. The Wilcoxon test showed no significant difference between the D_05_, D_95_ and UI values of the two groups of plans, implying that both sets of plans achieved similar coverage of the PTV.

### OAR sparing

**Fig. 1:**
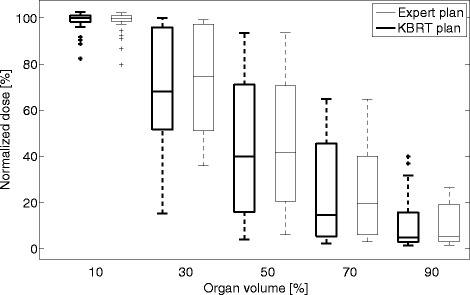
Bladder dose of the KBRT and expert plans. The central line of each box shows the median value, while the upper and lower edges represent the 25^th^ (Q1) and 75^th^ (Q3) percentiles respectively. The whiskers extend to values that are not considered as outliers (approximately mean ± 2.7 times standard deviation for normally distributed data). The individual pluses (+) are the extreme values that are considered as outliers. The Wilcoxon test shows that the KBRT plans achieved significantly lower dose to the bladder (at D_30_) when compared to the expert plans

**Fig. 2:**
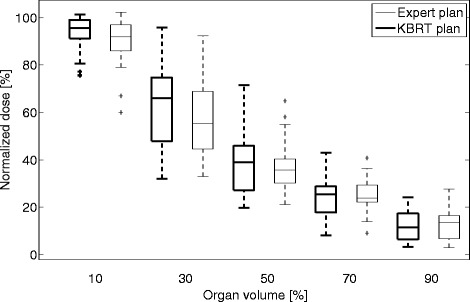
Rectal dose of the KBRT and expert plans. The Wilcoxon test results showed that the expert plans achieved significantly lower dose to the rectum (at D_30_ and D_10_) when compared to the KBRT plans

Figures 1 and 2 and Table 2 compare the planned dose to the bladder and rectum for the two approaches. Five dose-volume levels (D_10_, D_30,_ D_50_, D_70_ and D_90_) are used for the comparison. All values are normalized to the prescribed dose of the target volume. Table 2 provides a quantitative summary (average values and their standard deviations) of the results.

**Table 2:** Summary of the organ doses (mean ± standard deviation) of the KBRT and expert plans. All doses are normalized to dose that is prescribed to the target volume. The Wilcoxon test shows significantly lower bladder dose for the KBRT plans (at D_30_), as well as lower rectal dose (at D_10_ and D_30_) for the expert plans

Organ volume [%]	Bladder			Rectum		
	Mean organ dose of KBRT plans [%]	Mean organ dose of expert plans [%]	Mean organ dose difference (KBRT - expert)	Mean organ dose of KBRT plans [%]	Mean organ dose of expert plans [%]	Mean organ dose difference (KBRT- expert)
10	98.4 ± 4.4	98.3 ± 5.0	0.1 ± 2.9	93.0 ± 7.9	90.1 ± 9.5	3.0 ± 4.5
30	70 ± 25	74 ± 22	−3.0 ± 6.3	63 ± 17	57 ± 16	5.6 ± 8.1
50	44 ± 30	46 ± 29	−2.7 ± 9.1	39 ± 13	36.8 ± 9.9	2.4 ± 7.5
70	24 ± 22	24 ± 19	0.0 ± 7.1	24.6 ± 9.0	24.8 ± 7.1	−0.3 ± 5.4
90	11 ± 12	9.8 ± 8.7	1.0 ± 6.2	11.9 ± 6.2	12.6 ± 6.8	−0.7 ± 3.4

Figure 1 shows the planned dose to bladder of the KBRT and expert plans. The KBRT plans achieved a marginally lower dose to the bladder compared to the expert plans.

Figure 2 compares the planned dose to rectum of the KBRT and expert plans. The expert plans achieved lower rectal dose compared to the KBRT plans.

## Discussion

In this work the treatment plan qualities of two sets of plans that are generated by an experienced planner and a novice are investigated. The novice planner was guided by a knowledge-based radiation therapy (KBRT) treatment planning algorithm [2]. The results show that both sets of plans achieved similar target coverage. The KBRT approach achieved an overall statistically significant lower bladder dose compared to the expert plans, while the expert plans achieved a statistically significant lower rectal dose compared to the KBRT plans.

Considering the magnitude of the difference between the mean OAR doses (Table 2), it can be argued that the expert plans are superior. However, this is not a statement of inferiority of the objective approach, but may be suggestive of the need to improve the organ model of the rectum in the KBRT algorithm. The model can be improved for example through the careful selection of the data that are included in the learning database as discussed in the previous publication [2]. Although the Wilcoxon test show statistically significant differences in the OAR doses of the two approaches, it is unclear if these differences are clinically relevant.

Earlier studies suggest that KBRT plans are non-inferior to those that are made by experienced planners [12, 1]. Even if marginally inferior to the subjective approach, the objective KBRT approaches offer some advantages over the subjective approach (lower number of replans, less dependence on the experience of the planner, reduced planning time, etc.). This study thus shows that KBRT planning is capable of reducing inter-personnel variability of treatment plan quality [9] and also demonstrates that the influence of the experience of the planner can be minimized with the aid of an optimized KBRT algorithm. A prototype of the algorithm for clinical use is planned in the future after further validation of the algorithm for other treatment sites.

## Conclusion

A KBRT algorithm for the prostate was validated using treatment plans of 30 patients with prostate carcinoma. The results of our study suggest that KBRT treatment planning based on our published algorithm is a valid method to objectively include individual patient anatomical information in the treatment planning process. The results show that treatment plan quality achieved with the KBRT approach is comparable to the plan quality of the experienced planners.

## Abbreviations

KBRT: Knowledge-based radiation therapy treatment planning
OAR: Organ at risk
UI: Uniformity index
PTV: Planning target volume
VMAT: Volumetric modulated arc therapy
CT: Computed tomography
